# Diagnostic value of IMP3 and p53 immunohistochemical staining in EUS-guided fine-needle aspiration for solid pancreatic tumors

**DOI:** 10.1038/s41598-021-96492-4

**Published:** 2021-08-26

**Authors:** Rintaro Mikata, Shin Yasui, Takashi Kishimoto, Yusuke Kouchi, Ayako Shingyoji, Junichi Senoo, Koji Takahashi, Hiroki Nagashima, Yuko Kusakabe, Hiroshi Ohyama, Izumi Ohno, Harutoshi Sugiyama, Tetsuhiro Chiba, Jun Kato, Naoya Kato

**Affiliations:** 1 Department of Gastroenterology, Chiba University Graduate School of Medicine, 1-8-1 Inohana, Chuo-ku, Chiba, 260-8670 Japan; 2 Department of Molecular Pathology, Chiba University Graduate School of Medicine, Chiba, Japan

**Keywords:** Cancer, Gastrointestinal cancer

## Abstract

We previously identified insulin-like growth factor-II messenger ribonucleic acid-binding protein 3 (IMP3) as a valuable marker to distinguish malignant from benign lesions in pancreatic solid masses. The aim of this prospective study was to evaluate the usefulness of IMP3 and p53 immunohistochemical staining in endoscopic ultrasound-guided fine-needle aspiration (EUS–FNA) samples for pancreatic solid masses. The study recruited 90 consecutive patients with pancreatic masses, including 62 pancreatic ductal adenocarcinomas (PDACs), 11 benign tumors, and 17 other tumors, who underwent EUS–FNA, and conducted IMP3 and p53 immunohistochemical staining. The main outcome measurement was improved diagnostic utility using IMP3 and p53 immunohistochemical staining. IMP3 and p53 expressions were detected in 60.8% and 49.4% of malignant lesions, 69.4% and 58.1% of PDACs, and 0% of benign lesions, respectively. In PDAC and benign tumors, the use of IMP3 and/or p53 immunostaining increased the sensitivity of cytohistological analysis from 88.7 to 93.5%, although the difference was not statistically significant. The sensitivity of histological analysis combined with that of IMP3 staining was 91.9%, which was significantly greater than that of histology alone (80.6%). The use of IMP3 and p53 immunohistochemical staining did not significantly improve the sensitivity of cytohistological analysis; however, IMP3 staining may be helpful for the histological analysis of malignant pancreatic tumors.

## Introduction

Pancreatic ductal adenocarcinoma (PDAC) is one of the most aggressive malignant tumors, with a 5-year survival rate of only 4%^1^. Due to increasing incidence and death rates, PDAC is predicted to become the second leading cause of cancer-related deaths in 2020 in the US^2^. Endoscopic ultrasound-guided fine-needle aspiration (EUS–FNA) is a safe and effective technique that can be used to diagnose pancreatic tumors, with a pooled sensitivity of 85%–92% and specificity of 96%–98%^3–5^. However, differentiating between malignant and benign tumors can be challenging due to small specimens obtained by EUS–FNA. The use of various new technical methods, new devices, and molecular analysis has been reported to improve the sensitivity and accuracy of EUS–FNA for pancreatic tumors^6–9^. Two novel needles (Franseen and fork-tip needles) were recently developed for performing EUS-guided fine-needle biopsies^9^. KRAS mutation analysis was reported to be a useful tool in a prospective study and meta-analysis^8,10^, and high sensitivity was reported using next-generation sequencing for EUS–FNA samples^11^. Immunohistochemical staining for EUS–FNA samples is easily performed in daily medical practice; however, only one prospective study has investigated its diagnostic utility in pancreatic cancer^12^.

Recently, we retrospectively reported that insulin-like growth factor-II messenger ribonucleic acid-binding protein 3 (IMP3) is a valuable marker to distinguish malignant from benign lesions in pancreatic solid masses^13^. IMP3 is an oncofetal protein that is expressed during embryogenesis and is almost silenced in normal mature tissues. IMP3 may play an important role in mRNA trafficking and stabilization, localization, cell growth, and cell migration^14^. It has also been reported to be frequently expressed in many different tumor types and is typically associated with aggressive tumor features^15^. Overexpression of IMP3 in PDAC has also been reported^16–21^ and was recently reported to be significantly associated with poor prognosis; therefore, it may be a potential therapeutic target for PDAC^18^.

Some retrospective studies have reported IMP3 expression as a valuable marker to distinguish PDAC from benign lesions using histological samples obtained using a core biopsy needle or cytological samples obtained using an FNA needle^16,22^. However, there have been no prospective studies investigating whether cytohistological analysis combined with IMP3 expression can significantly improve the diagnostic value in pancreatic masses. The p53 tumor suppressor gene is significantly mutated in many tumors, including human pancreatic cancer (38.2%–81.1%)^12,23–25^. Hence, immunohistochemical staining of p53 could improve the diagnostic accuracy of PDAC.

The present prospective study evaluated the diagnostic value of IMP3 and p53 in EUS–FNA specimens of solid pancreatic masses.

## Methods

Consecutive patients referred for EUS–FNA of pancreatic masses were prospectively enrolled from December 2015 to December 2016. Written informed consent was obtained from all participants. The inclusion criteria were as follows: age of > 20 years; presence of a solid pancreatic mass confirmed by computed tomography scan, magnetic resonance imaging, abdominal ultrasound, or EUS; and ability to provide written informed consent. The exclusion criteria were as follows: poor general condition, presence of a known bleeding disorder or massive ascites, pregnancy, and inability to sample the lesion due to the presence of intervening blood vessels. A total of 90 patients [males, females; median age, 65.8 ± 11.5 (range: 34–88) years] were prospectively enrolled in this study. The clinical data collected included age, sex, tumor size, location, puncture route, number of needle passes, and needle size (Table 1). The final diagnosis was based on the histological analysis of surgically resected specimens and cytopathological detection of cancer cells from other organs coupled with clinical and/or radiological evidence of progressive disease. Benign lesions were diagnosed in surgically resected specimens or during clinical follow-up of at least 12 months, with no evidence of progressive disease. Autoimmune pancreatitis was diagnosed according to the Japanese Clinical Diagnostic Criteria for Autoimmune Pancreatitis, 2011, and the pathological results of EUS-FNA were included in the assessment^26^. The study protocol was approved by the Institutional Ethics Review Board of Chiba University Hospital, Japan, and was registered in the University Hospital Medical Information Network with the identifier UMIN000020223. All methods were performed in accordance with the relevant guidelines and regulations.

**Table 1:** Patients’ characteristics.

Number of patients (M/F)	90 (58/32)
Type of tumor (PDAC/other tumors/benign tumor)	62/17/11
Mean age, years (± SD) (range)	65.8 ± 11.5 (34–88)
Location of mass (head/body/tail)	46/31/13
Mean size of mass, mm (± SD) (range)	26.5 ± 11.4 (7.2–53.3)
Puncture route (stomach/duodenum/other)	53/36/1
Mean number of needle passes (± SD)	2.88 ± 0.9
Needle size (22 G/25 G/other)	60/29/1

### EUS–FNA procedure

EUS–FNA was performed using a 19, 22, or 25-gauge SonoTip (Medi-Globe) or Expect (Boston Scientific) needle with an average of 2.9 ± 0.9 passes per session (Table 1). The aspirated biopsy material was pushed onto a filter paper by re-inserting the stylet into a formalin-filled container for histological analysis. The residual material was smeared onto a glass slide using air pressure and fixed with 95% ethanol for cytological analysis. Puncture was repeated until sufficient material was confirmed by Rapid on-site evaluation (ROSE). ROSE results were available for all cases in this study. Formalin-fixed biopsy specimens were embedded in paraffin and stained with hematoxylin and eosin (HE) for histological analysis. For histological analysis, the specimens were classified into five types: malignancy, suspected malignancy, atypical cells, no evidence of malignancy, or insufficient material. Specimens classified as malignancy and suspected malignancy by HE staining were defined as positive for malignancy, whereas those classified as atypical cells, no evidence of malignancy, and insufficient materials were defined as negative for malignancy.

### Immunohistochemical staining of EUS–FNA specimens

IMP3 immunohistochemistry was performed using mouse monoclonal anti-human IMP3 (clone 69.1; Dako, Glostrup, Denmark) and mouse monoclonal anti-human p53 protein (Clone DO7; Dako) primary antibodies diluted 1:100 and 1:50, respectively.

**Figure 1:**
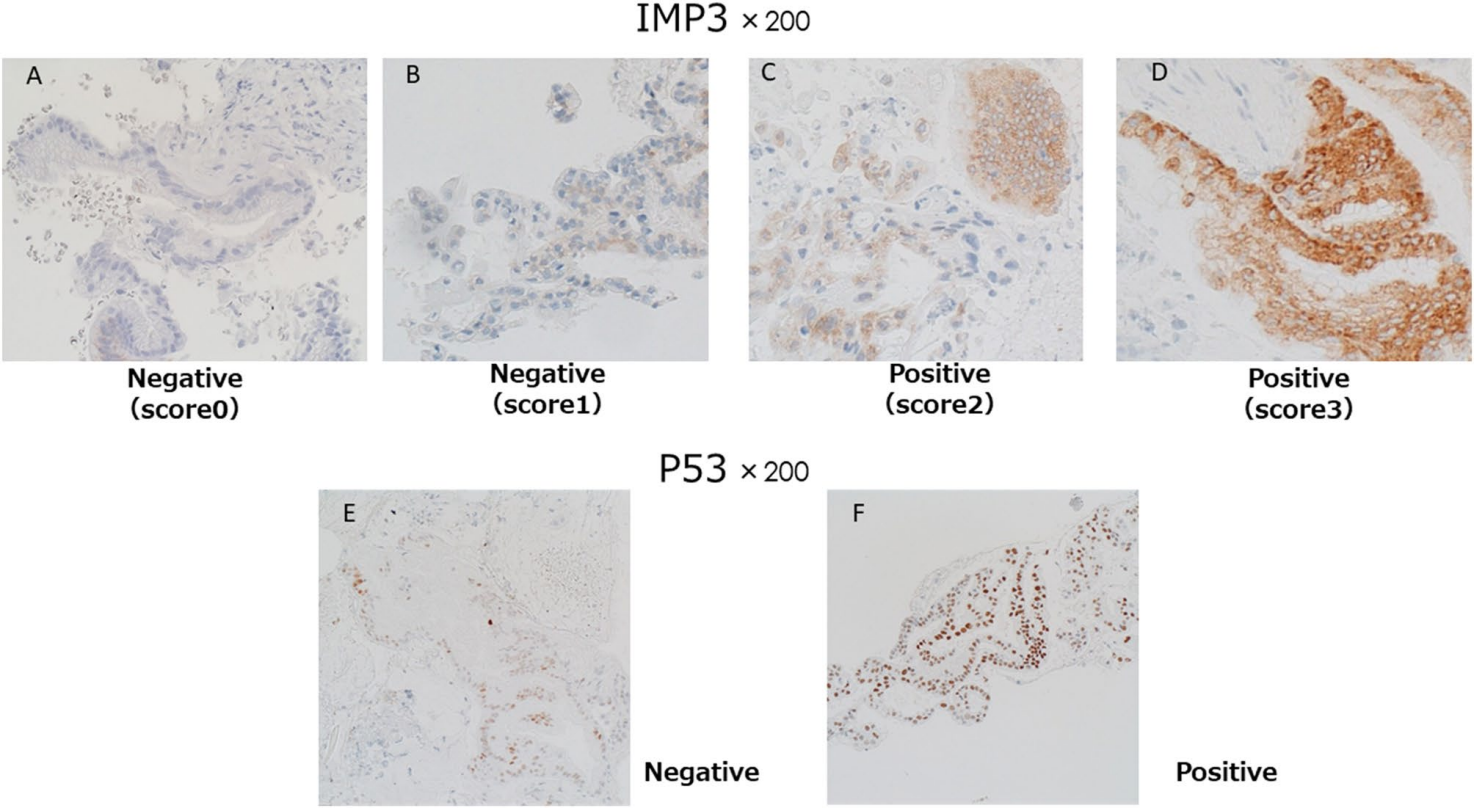
Representative positive and negative cases for IMP3 and TP53. Immunohistochemical IMP3 expression revealed negative (score 0) staining in (**A**), negative (score 1+) staining in (**B**), moderate (score 2+) staining in (**C**), strong (score 3+) staining in (**D**). Immunohistochemical P53 expression revealed negative staining in (**E**) and positive staining in (**F**).

**Figure 2:**
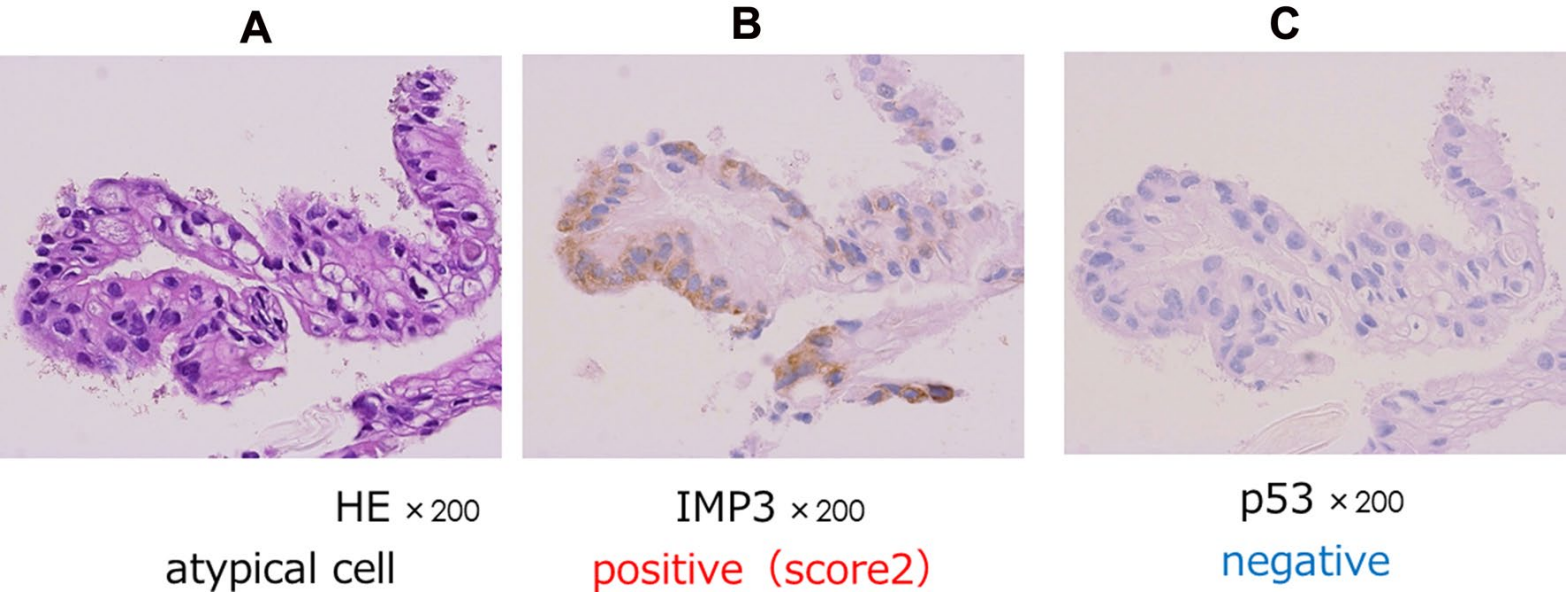
Representative case of pancreatic cancer. (**A**) Hematoxylin and eosin (HE) staining revealed an atypical epithelium with a small amountof tissue, (**B**) Immunohistochemistry for IMP3 revealed moderate (score 2+) staining. (**C**) Immunohistochemistry for P53 was negative.

IMP3 staining was evaluated using a four-tiered system for staining intensity, as previously described^26^. When at least 10% of cells were stained positive, the staining intensity was scored as 0 (negative), 1 + (weak), 2 + (moderate), or 3 + (strong)^27^. Scores of 0 or 1 + were considered negative for IMP3, whereas scores of 2 + or 3 + were considered positive. Abnormal p53 labeling was defined as positive nuclear staining of at least 60% of tumor cells^28,29^. Representative images of immunohistochemical IMP3 and P53 expression are shown in Fig. 1 and representative cases of pancreatic cancer are shown in Fig. 2. EUS–FNA specimens positive for IMP3 or p53 were considered positive for malignancy. All slides with EUS–FNA samples were stained with HE and reviewed by the same experienced pathologist (T.K.). In this prospective study, the immunohistochemical results were reviewed by only one pathologist (T.K.). In addition, another pathologist (Y.K.) reviewed the immunohistochemical results of the same specimens independently, and their concordance was also retrospectively confirmed.

### Quantification of EUS–FNA samples

The amount of sample obtained via EUS–FNA was assessed by a pathologist (T.K.) using the modified scoring system previously described by Gerke et al.^30^. The scoring system was as defined follows: 0, insufficient amount of material; 1, sufficient material for limited cytological interpretation: not representative; 2, sufficient material for adequate cytological interpretation; 3, sufficient material for limited histological interpretation; and 4, sufficient material for adequate histological interpretation. The relationship between immunohistochemical expression and tissue quantity was evaluated.

### Sample size calculation

The sample size was determined as described in our previous study^13^. An 80% accurate diagnosis was confirmed by histocytological analysis and 90% was confirmed by histocytological plus immunohistological analyses. Based on this, we calculated that a sample size of 90 cases was required to detect a difference of 10 percentage points using the McNemar test, assuming 10% drop out of the enrolled patients, with a power of 80% using a two-sided significance rate of 5%.

### Statistical analysis

Statistical analysis was performed using SPSS version 22 (SPSS Inc., Chicago, IL, USA). Quantitative data are presented as mean ± standard deviation. Analysis of EUS–FNA specimens used the McNemar test to assess the statistical significance of sensitivity, specificity, and accuracy comparing cytology and histology alone as well as a combination of cytology, pathology, and immunohistochemical staining. *P*-values of < 0.05 were considered statistically significant.

## Results

### EUS–FNA staining

Among 90 EUS–FNA solid pancreatic mass specimens, 79 were malignant and 11 were benign. Malignant lesions included 62 PDACs, 6 neuroendocrine tumors (NETs), 3 malignant intraductal papillary-mucinous neoplasms, 2 solid-pseudopapillary neoplasms (SPNs), 1 neuroendocrine carcinoma, 1 acinar cell carcinoma, 1 schwannoma, 1 gastrointestinal stromal tumor (GIST), for which the final diagnosis was duodenal GIST and not an intrapancreatic GIST, 1 mixed neuroendocrine non-neuroendocrine neoplasm (MiNEN), and 1 metastatic tumor of primary unknown carcinoma. For the malignant lesions, the diagnoses of 25 patients with PDAC and all other malignant lesions except for one metastatic tumor of unknown primary carcinoma were based on the histological analysis of surgically resected specimens. The other 38 patients were diagnosed based on the cytopathological detection of cancer cells from other organs coupled with clinical and/or radiological evidence of progressive disease; the mean follow-up period of these patients was 361 days. The benign lesions included seven cases with autoimmune pancreatitis, three with focal chronic pancreatitis, and one intraductal papillary-mucinous adenoma (IPMA) (Table 2). IPMA was diagnosed in surgically resected specimens, whereas the other benign lesions were diagnosed by EUS-FNA with a clinical follow-up of at least 12 months with no evidence of progressive disease; the mean follow-up period of these patients was 628 days. Seven patients with autoimmune pancreatitis were diagnosed according to the Japanese Clinical Diagnostic Criteria for Autoimmune Pancreatitis, 2011.

**Table 2:** Final diagnosis.

PDAC	62
**Benign diseases**	
Focal chronic pancreatitis	3
Autoimmune pancreatitis	7
IPMA	1
**Other malignant tumors**	
PNET	6
IPMC	3
SPN	2
Neuroendocrine carcinoma	1
Acinar cell carcinoma	1
Schwannoma	1
GIST	1
MANEC	1
Primary unknown	1

GIST, gastrointestinal stromal tumor; IPMA, intraductal papillary-mucinous adenoma; IPMC, intraductal papillary-mucinous carcinoma; MANEC, mixed adenoneuroendocrine carcinoma; PNET, pancreatic neuroendocrine tumor; SPN, solid-pseudopapillary neoplasm.

Immunohistochemical staining was performed and evaluated in 88/90 specimens, but could not performed for two specimens due to insufficient sample size in one case of SPN and one of schwannoma.

IMP3-positive expression was observed in 47/79 (60.8%) malignant cases, including 43/62 (69.4%) PDACs, 5/17 other malignant tumors, and 0/11 benign tumors (Tables 3 and 4). One grade 2 NET, one neuroendocrine carcinoma (NEC), and one MiNEN case showed IMP3-positive expression, whereas the other five NET cases showed IMP3-negative expression. Furthermore, 2/3 intraductal papillary-mucinous carcinoma (IPMC) cases were IMP3-positive. IMP3 expression was negative in all benign lesion specimens. Overexpression of p53 was observed in 39/79 (49.4%) malignant cases including 36/62 (58.1%) PDACs, 3/17 other malignant tumors, and 0/11 benign tumors. Moreover, 1/3 IPMC cases, one NEC case, and one GIST case were p53-positive.

**Table 3:** Results of EUS–FNA (all cases).

	Sensitivity (%)	Specificity (%)	Accuracy (%)
IMP3	60.8 (48/79)	100 (11/11)	65.6 (59/90)
P53	49.4 (39/79)	100 (11/11)	55.6 (50/90)
IMP3 or p53	74.7 (59/79)	100 (11/11)	77.8 (70/90)
Histology	78.5 (62/79)	90.9 (10/11)	80.0 (72/90)
Cytology	64.6 (51/79)	100 (11/11)	68.9 (62/90)
Histology + Cytology	84.8 (67/79)	90.9 (10/11)	85.6 (77/90)
Histology + IMP3	88.6*^1^ (70/79)	90.9 (10/11)	88.9*^1^(80/90)
Histology + p53	84.8 (67/79)	90.9 (10/11)	85.6 (77/90)
Histology + Cytology + IMP3	88.6 (70/79)	90.9 (10/11)	88.9 (80/90)
Histology + Cytology + p53	87.3 (69/79)	90.9 (10/11)	87.8 (79/90)
Histology + Cytology + IMP3 + p53	89.9*^2^ (71/79)	90.9 (10/11)	90.0*^2^(81/90)

^1^*P* = 0.01, Histology vs. Histology + IMP3.

^2^*P* = 0.13, Histology + Cytology vs. Histology + Cytology + IMP3 + p53.

EUS–FNA, endoscopic ultrasound-guided fine-needle aspiration.

**Table 4:** Results of EUS–FNA (PDAC and benign diseases).

	Sensitivity (%)	Specificity (%)	Accuracy (%)
IMP3	69.4 (43/62)	100 (11/11)	74.0 (54/73)
P53	58.1 (36/62)	100 (11/11)	64.4 (47/73)
IMP3 or p53	83.9 (52/62)	100 (11/11)	86.3 (63/73)
Histology	80.6 (50/62)	90.9 (10/11)	82.2 (60/73)
Cytology	72.6 (45/62)	100 (11/11)	76.7 (56/73)
Histology + Cytology	88.7 (55/62)	90.9 (10/11)	89.0 (65/73)
Histology + IMP3	91.9*^1^ (57/62)	90.9 (10/11)	91.8*^1^ (67/73)
Histology + p53	88.7 (55/62)	90.9 (10/11)	89.0 (65/73)
Histology + Cytology + IMP3	91.9 (57/62)	90.9 (10/11)	91.8 (67/73)
Histology + Cytology + p53	91.9 (57/62)	90.4 (10/11)	91.8 (67/73)
Histology + Cytology + IMP3 + p53	93.5*^2^ (58/62)	90.9 (10/11)	93.2*^2^ (68/73)

^1^*P* = 0.02, Histology vs. Histology + IMP3.

^2^*P* = 0.25, Histology + Cytology vs. Histology + Cytology + IMP3 + p53.

EUS–FNA, endoscopic ultrasound-guided fine-needle aspiration.

The sensitivity, specificity, and accuracy of cytohistology combined with IMP3 and p53 immunohistochemistry were 89.9%, 90.9%, and 90.0%, respectively, for all cases (Table 3). Except for the two cases in which insufficient data were available, these sensitivity, specificity, and accuracy were 92.2%, 90.9%, and 92.0%, respectively. The sensitivity, specificity, and accuracy of PDACs and benign diseases were 93.5%, 90.9%, and 93.2%, respectively (Table 4). The sensitivity of cytohistological analysis combined with IMP3 and p53 staining was not significantly greater than that of cytohistology alone either for all cases or PDACs and benign diseases (84.8% vs. 89.9% for all cases,* P* = 0.13; and 88.7% vs. 93.5% for PDACs and benign diseases, *P* = 0.25). However, the sensitivity of histological analysis combined with IMP3 staining was significantly greater than that of histology alone for all cases and for PDACs and benign diseases (78.5% vs. 88.6% for all cases, *P* = 0.01; 80.6% vs. 91.9% for PDACs and benign diseases, *P* = 0.02). The accuracy of cytohistological analysis combined with IMP3 and p53 staining was not significantly greater than that of cytohistology alone for all cases of PDACs and benign diseases. However, the accuracy of histological analysis combined with IMP3 staining was significantly greater than that of histology alone for all cases of PDACs and benign diseases.

The present study identified one false-positive case with a benign tumor that was diagnosed as pancreatic adenocarcinoma in histological examination prior to surgery, but diagnosed as IPMA after surgery. This IPMA lesion did not have a cystic lesion and appeared like a mass lesion, possibly because inflammation was confirmed by histological analysis. Therefore, the specificity was slightly decreased.

The amount of sample obtained using EUS–FNA was assessed using the scoring system previously described by Gerke et al.^30^. The mean scores were 3.45, 3.71, and 3.18 for PDACs, other tumors, and benign diseases, respectively (Table 5). The scores for benign diseases tended to be lower than those for other tumors (*P* = 0.07). In PDAC, the mean scores were 3.72, 3.00, and 2.91 for the diagnoses of malignancy, suspicious malignancy, and atypical cells (Table 6). The mean scores for cases with atypical cells were significantly lower than those for cases with malignancy (*P* = 0.005).

**Table 5:** Tissue amount assessed using scoring system for PDAC, others, and benign diseases.

	Score mean (± SD)	Score ≥ 3
All (n = 90)	3.52 ± 1.03	77 (85.6%)
PDAC (n = 62)	3.45 ± 0.97	54 (87.1%)
Others (n = 17)	3.71 ± 1.57	14 (82.4%)
Benign diseases (n = 11)	3.18 ± 1.18	9 (81.8%)

**Table 6:** Tissue amount assessed using scoring system according to histological results of PDAC.

	Score mean (± SD)	Score ≥ 3
**PDAC (n = 62)**		
Malignancy (n = 47)	3.72 ± 0.82	43 (91.5%)
Suspicious of malignancy (n = 3)	3.00 ± 1.00	2 (66.7%)
Atypical cells (n = 12)	2.91 ± 0.79*^1^	8 (66.7%)

^1^*P* = 0.005, Malignancy vs atypical cells.

Among the PDAC specimens diagnosed as malignancy or suspected malignancy by HE staining, 72% (36/50) were positive for IMP3 staining. In addition, 7/12 (58.3%) samples diagnosed as atypical cells were IMP3-positive, whereas 5/12 (41.7%) samples diagnosed as atypical cell were p53-positive (Table 7).

**Table 7:** Immunohistochemical staining in PDAC according to histological result.

	n	IMP3		p53		
		Positive	%	Positive		%
Malignancy	47	35	74.5	29		61.7
Suspected malignancy	3	1	33.3	2		66.7
Atypical cells	12	7	58.3	5		41.7
Total	62	43	69.4	36		58.1

Analysis of the relationship between immunohistochemical staining and amount of tissue in PDAC revealed IMP3 positivity in 25/35 (71.4%) grade 4 or 5 specimens, 10/18 (55.6%) grade 3 specimens, and 8/9 (88.9%) grade 2 specimens, whereas p53 positivity was observed in 21/35 (60%) grade 4 or 5 specimens, 9/18 (50%) grade 3 specimens, and 6/9 (66.7%) grade 2 specimens. Tissue amount was not related to combined IMP3 and p53 immunohistochemical staining (Table 8).

**Table 8:** Immunohistochemical staining in PDAC according to tissue amount.

Grade	n	IMP3		p53		
		Positive	%	Positive		%
2	9	8	88.9	6		66.7
3	18	10	55.6	9		50
4 or 5	35	25	71.4	21		60
Total	62	43	69.4	36		58.1

Nine patients with PDAC had a score of 2 (Table 8). Among them, six patients were diagnosed with adenocarcinoma (four patients) or were suspected to have adenocarcinoma (two patients) because the pathologist could make a diagnosis based on cytological atypia using specimens prepared for histological analysis. Of the other three patients with atypical cells, two were IMP3-positive, which led to the diagnosis of malignancy.

The concordance rates of IMP3 and P53 positivity between the two pathologists were reviewed. As for IMP3 staining, one pathologist (T.K.) classified 32 specimens as 0, 8 as 1+ , 19 specimens as 2+ , and 29 as 3+ . The other pathologist (Y.K.) classified 23 specimens as 0, 13 as 1+ , 23 as 2+ , and 29 as 3+ . IMP3 positivity was concordant in 84 (95.4%) of 88 specimens between the two pathologists. In all four inconsistent cases, one pathologist (T.K.) classified the specimens as 1+ , whereas the other pathologist (Y.K.) classified them as 2+ . As for P53, p53 positivity was concordant in 85 (96.6%) of 88 specimens between the two pathologists.

## Discussion

The results of the present study revealed that the sensitivity and accuracy of cytohistological analysis combined with IMP3 and p53 staining were not significantly greater than those of cytohistology alone. However, the sensitivity and accuracy of histological analysis combined with IMP3 staining were significantly greater than those of histology alone in all cases as well as cases of PDACs and benign diseases. Therefore, IMP3 staining may be a valuable marker for the histological differentiation of malignant from benign pancreatic disease.

It is important to establish a pathological diagnosis prior to the treatment of PDAC. EUS–FNA is increasingly used worldwide to obtain tumor samples. However, diagnosis of PDAC may be difficult because of the small amount of tissue sample obtained. In such cases, gene mutation analysis or immunohistochemical markers may help differentially diagnose malignant from benign lesions. KRAS mutation analysis has been reported to be a useful tool for the differential diagnosis of pancreatic masses. However, it was reported to have 3–32% false-positive rate in benign masses^8,31^, and immunohistochemical analysis is easier to perform in daily clinical practice.

IMP3 has been reported as a useful diagnostic marker of PDAC for the analysis of core needle biopsy samples or cytological samples^16,22^. We recently retrospectively reported that IMP3 was a valuable marker to distinguish malignant from benign lesions for pancreatic solid masses in samples obtained by EUS–FNA as well as surgical samples^13^. In these studies, IMP3 was reported to be diffusely expressed in the entire tumor; therefore, it was useful for analyzing small specimens obtained by EUS–FNA. In our retrospective study, the tissue amount was correlated with IMP3 positivity. However, in the present study, we showed that tissue amount was not related to IMP3 immunohistochemical staining. Furthermore, 8/9 grade 2 PDAC specimens were positively diagnosed. Although the reason for this discrepancy is unknown, the results of this study were more plausible because IMP3 staining was believed to be positive even in small samples, if the tumor expressed any IMP3.

In the present study, the sensitivity of the combination of cytohistology and IMP3 and p53 for PDAC and benign diseases improved to 93.5% from 88.7% of cytohistology alone; however, this difference was not statistically significant. In our retrospective study, the sensitivity of the combination of cytohistology and IMP3 and p53 for PDAC and benign diseases significantly improved to 90.4% from 81.9% of cytohistology alone. In a retrospective study, EUS–FNA samples stained with HE and immunohistochemical slides were reviewed by three pathologists, including non-experts in pancreaticobiliary diseases. In this prospective study, all EUS–FNA samples were reviewed by the same experienced pathologist who was an expert in pancreaticobiliary diseases. Therefore, the histological results obtained by HE staining alone were approximately 10% better in the present study than in the retrospective studies. This may explain the differences in results seen between these retrospective and prospective studies.

Histological analyses revealed that the sensitivity of histology combined with IMP3 staining significantly improved from 78.5% and 80.6% of histology alone to 88.6% and 91.9% for all cases and PDAC and benign diseases, respectively. Diagnoses of “atypical cell” were confirmed in 24.4% (22/90) by cytology and 15.6% (14/90) by histology. Twelve cases were histologically diagnosed as atypical cell mainly due to the small sample sizes; however, 7/12 (58.3%) PDAC cases were IMP3-positive. These findings were similar to those found in our retrospective study, in which 12/16 (75.0%) PDAC cases histologically diagnosed as atypical cell were IMP3-positive. Therefore, IMP3 staining should be considered valuable to distinguish malignant from benign lesions in small tissue samples that are difficult to evaluate by HE staining alone. This may be particularly useful in institutions that lack an expert pathologist of pancreaticobiliary diseases.

In the present study, the sensitivity and accuracy of p53 immunohistochemical staining of EUS–FNA specimens were not significantly improved compared with those of routine analysis, which is the same as those seen in our retrospective study. The criteria for p53 expression were strict to avoid false-positive diagnoses, according to previous reports^23,29^. These strict criteria may result in relatively low positivity for p53 in malignant tumors and PDAC. However, p53 immunohistochemical staining in combination with routine analysis may help enhance confidence in diagnoses.

The strength of our study is that this is the first prospective study to evaluate the diagnostic value of IMP3 and p53 immunohistochemical analysis for solid pancreatic tumors. In our previous retrospective study, IMP3 and p53 immunohistochemical analysis was performed in only 105 of 125 specimens due to insufficient material for histological examination or because the corresponding paraffin sections did not contain sufficient material to perform immunohistochemical analysis, which might have caused selection bias. In this prospective study, we were able to perform a histological examination in 88 of 90 cases but not in 2 cases in which insufficient material was present; we also performed immunohistochemistry in all 88 cases.

The present study has some limitations. First, this was a single-center study. Second, all EUS–FNA samples were only reviewed by one experienced pathologist who was expert in pancreaticobiliary diseases, and diagnoses were not based on the consensus of more than two pathologist. Third, there were only 11 cases with benign diseases; therefore, it is unclear whether there was false-positive staining for IMP3 or p53.

## Conclusions

In conclusion, IMP3 and p53 immunohistochemical staining did not significantly improve the sensitivity of cytohistological analysis; however, the results of the present study demonstrate the usefulness of IMP3 as an immunohistochemical marker for histological analysis of solid pancreatic masses obtained by EUS–FNA.

## Abbreviations

HE: Hematoxylin and eosin
IPMA: Intraductal papillary-mucinous adenoma
IPMC: Intraductal papillary-mucinous carcinoma
MiNEN: Mixed neuroendocrine non-neuroendocrine neoplasm
NEC: Neuroendocrine carcinoma
SPN: Solid-pseudopapillary neoplasms
EUS–FNA: Endoscopic ultrasound-guided fine-needle aspiration
GIST: Gastrointestinal stromal tumor
MANEC: Mixed adenoneuroendocrine carcinoma
PDAC: Pancreatic ductal adenocarcinomas
PNET: Pancreatic neuroendocrine tumor
